# Prevalence, predictors and prognosis of incidental intracranial aneurysms in patients with suspected TIA and minor stroke: a population-based study and systematic review

**DOI:** 10.1136/jnnp-2020-324418

**Published:** 2020-11-04

**Authors:** Robert Hurford, Isabel Taveira, Wilhelm Kuker, Peter M Rothwell, the Oxford Vascular Study Phenotyped Cohort

**Affiliations:** Centre for the Prevention of Stroke and Dementia, University of Oxford, Oxford, Oxfordshire, UK

## Abstract

**Introduction:**

Unruptured intracranial aneurysms (UIAs) are common incidental imaging findings, but there are few data in patients with transient ischaemic attack (TIA)/stroke. The frequency of UIA might be higher due to shared risk factors, but rupture risk might be reduced by intensive secondary prevention. We determined the prevalence and prognosis of UIA in patients with suspected TIA/minor stroke.

**Methods:**

All patients referred to the population-based Oxford Vascular Study (2011–2020) with suspected TIA/minor stroke and non-invasive angiography were included. We determined the prevalence of incidental asymptomatic UIA and the risk of subsequent subarachnoid haemorrhage (SAH) by follow-up on intensive medical treatment, with guideline-based monitoring/management. We also did a systematic review of UIA prevalence/prognosis in cohorts with TIA/stroke.

**Findings:**

Among 2013 eligible patients, 95 (4.7%) had 103 previously unknown asymptomatic UIA. Female sex (OR 2.3, 95% CI 1.5 to 3.7), smoking (2.1, 1.2 to 3.6) and hypertension (1.6, 1.0 to 2.5) were independently predictive of UIA, with a prevalence of 11.1% in those with all three risk factors. During mean follow-up of 4.5 years, only one SAH occurred: 2.3 (95% CI 0.3 to 16.6) per 1000 person-years. We identified 19 studies of UIA in TIA/stroke cohorts (n=12 781), all with either symptomatic carotid stenosis or major acute stroke. The pooled mean UIA prevalence in patients with TIA/stroke was 5.1% (95% CI 4.8 to 5.5) and the incidence of SAH was 4.6 (95% CI 1.9 to 11.0) per 1000 person-years.

**Interpretation:**

The 5% prevalence of UIA in patients with confirmed TIA/minor stroke is likely higher than that in the general population. However, the risk of SAH on intensive medical treatment and guideline-based management/monitoring is low.

## Introduction

Saccular unruptured intracranial aneurysms (UIAs) are present in 3% of the adult general population^1^ and are the most common incidental finding on neuroimaging in healthy individuals.^2^ Although the frequency and quality of non-invasive angiography in the assessment of suspected ischaemic stroke or transient ischaemic attack (TIA) is increasing, the optimal management of asymptomatic UIAs remains uncertain.^3^ Moreover, previous studies have been limited to selected cohorts under investigation for symptomatic carotid stenosis or major acute stroke.

Patients referred to acute neurovascular services for suspected TIA or minor stroke also have a greater burden of vascular risk factors and a higher susceptibility to cerebrovascular disease than the general population.^4^ As cerebrovascular disease and intracerebral aneurysm development have risk factors in common, it might be expected that these patients will also have a higher burden of UIAs. However, there have been no population-based studies assessing the prevalence of UIAs in a general cerebrovascular disease cohort and none of patients with TIA and minor stroke.

Furthermore, while there may be a higher prevalence of UIAs in a cerebrovascular disease cohort, it is unknown whether the risk of rupture is comparable with that in the general population. Risk factors for intracerebral aneurysm rupture, such as hypertension and smoking, are common but are more intensively managed after TIA or ischaemic stroke. Moreover, there is some evidence aspirin lowers the risk of intracerebral aneurysm rupture by reducing vessel wall inflammation.^5–7^ As patients with minor stroke and TIA would often be suitable candidates for intervention, the prognosis of incidental UIAs is of clinical importance.

We aimed to determine the prevalence and predictors of UIA in patients referred to an acute neurovascular service with suspected TIA or minor stroke, and to assess the risk of subarachnoid haemorrhage on follow-up with a standard guideline-based policy for intervention/surveillance. We also did a systematic review and meta-analysis of previous studies to establish the prevalence and prognosis of UIA in patients with TIA or stroke.

## Methods

The Oxford Vascular Study (OXVASC) is a longitudinal population-based incidence cohort of all acute vascular events in a defined population of 92 728, covered by around 100 primary care physicians in nine primary care practices in Oxfordshire, UK. An estimated 97% of the true study residential population is registered with a primary care practice; most unregistered people are young students. The study area contains a mix of urban and rural populations. The OXVASC population is 94% Caucasian, 3% Asian, 2% Chinese and 1% Afro-Caribbean.^8^

Detailed methodology of OXVASC has been reported previously.^9^ Multiple overlapping methods were used for ascertainment of all individuals with TIA and stroke, approaching 100% of events reaching medical attention. These include the following: (1) a daily, rapid access clinic to which participating primary care practitioners and the local emergency department refer individuals with suspected TIA or minor stroke; (2) daily searches of admissions to the medical, stroke, neurology and other relevant wards; (3) daily searches of the local emergency department attendance register; (4) monthly searches of general practitioner diagnostic coding and hospital discharge codes; (5) monthly searches of all brain and vascular imaging referrals. Patients gave written informed consent after an event or assent was obtained from a relative for patients who were unable to provide consent.^10 11^

We studied consecutive patients referred to OXVASC rapid access service between 1 March 2011 and 1 March 2020 with suspected TIA or minor ischaemic stroke (National Institute of Health Stroke Scale (NIHSS) score ≤3) and consenting to investigation. All patients with intracranial vascular imaging were included in the analyses.

Demographic data and stroke risk factors were collected from face-to-face interview by study physicians as soon as possible after referral or hospital admission and cross-referenced with primary care records. Detailed clinical history was recorded in all patients and assessments were made for stroke severity using the NIHSS as recorded on assessment. Cause of ischaemic events was classified according to the Trial of Org 10172 in Acute Stroke Treatment (TOAST) criteria.^11^ Stroke and TIA were defined according to WHO criteria (acute onset of neurological deficit, persisting for >24 hours in case of a stroke or for <24 hours in case of a TIA),^12^ with review of all cases as soon as possible after presentation by the same senior neurologist (PMR) throughout the study.

### Imaging procedures

Intracranial vascular imaging was done in all patients in OXVASC from 2011 onward. We aimed to obtain as high an imaging rate as possible by using MR angiography (MRA) as first choice and CT angiography (CTA; Toshiba, Aquilion 64, 64-slice scanner) if MRI was contraindicated (eg, implantable devices or claustrophobia).

The MRI scanners and protocols used in OXVASC have been described elsewhere,^13^ but sequences included diffusion-weighted imaging, time of flight angiography of the intracranial arteries and gadolinium contrast-enhanced angiography (CE-MRA) of the intracranial and cervicocranial arteries including the aortic arch. Patients were scanned at the Acute Vascular Imaging Centre, John Radcliffe Hospital, in a 3.0 T Siemens Verio scanner; a neurovascular coil was used (CE-MRA sequence: 15 mL ProHance followed by 40 mL NaCl, flow rate 2 mL/s, TR 22 ms, TE 3.6 ms, flip angle 18°, slice thickness 0.5 mm).

All imaging was reviewed and reported daily by a single study neuroradiologist (WK) and discussed at a weekly neuroradiology meeting. The number, location and size of UIA was recorded. Saccular aneurysms were defined as an abnormal swelling or bulge of an intracranial artery bifurcation with a distinct neck at the point of attachment to the parent vessel. Non-saccular aneurysms (fusiform aneurysms) were defined as a circumferential wall swelling or distention without a distinct neck; these are reported but not included in the analyses. The 5-year risk of aneurysm rupture was calculated using the PHASES score.^14^ The management of all incidental UIA was determined at a weekly multidisciplinary meeting of interventional neuroradiologists, neurologists and vascular neurosurgeons using a standard policy aligned to the American Heart Association/American Stroke Association guidelines for incidental UIA more generally.^15^

Patients were followed up face to face at 1, 6, 12, 24, 60 and 120 months by a study nurse or physician to identify any recurrent stroke (supplemented by review of primary care records) and to ensure medication compliance and adequate blood pressure control. Patients who had moved out of the study area (or were unwilling/unable to have face-to-face follow-up) were followed up via telephone at the same time points. All recurrent events, including subsequent subarachnoid haemorrhage (SAH), occurring during follow-up would also be identified by the ongoing daily case ascertainment. We recorded all deaths during follow-up with the underlying causes by direct follow-up, via primary care records, and by centralised registration with Office for National Statistics (ONS).

### Statistical analysis

Analyses included all eligible patients with intracranial vascular imaging. Baseline characteristics were compared between patients with and without UIA, subcategorised into discharge diagnosis, using χ^2^ or Student’s t-test as appropriate.

We calculated the age-specific prevalence of UIA in 10-year bands, stratified by sex. We determined the predictors of UIA with univariate and multivariate regression analyses.

We determined the absolute risk of subsequent SAH during follow-up (to 31 March 2020) after the index event and compared this with the predicted risk as calculated by the mean cohort PHASES score.

All statistical analyses were performed with IBM SPSS V.25.0.

### Systematic review

All studies reporting the prevalence of asymptomatic, saccular UIAs in patients with ischaemic stroke or TIA were included. Embase and Ovid MEDLINE databases were searched using the search terms (online supplemental figure 1) from inception until 1 May 2020. Intracranial vascular imaging by any modality was considered. Studies of patients with asymptomatic carotid stenosis only and case reports/series were excluded. The outcome of interest was the proportion of patients with UIAs and the outcome of the UIA. Disagreement over eligibility was resolved by discussion with the senior author (PMR).

10.1136/jnnp-2020-324418.supp1 Supplementary data

### Data abstraction and quality assessment

Key descriptive and quantitative data were recorded for study design, cohort characteristics and outcome data. Recorded details included the year of study publication, study location number of participants, proportion of females, mean cohort age, imaging modality, the prevalence of UIAs and UIA outcome if reported.

### Meta-Analysis

The leading outcome of interest was the prevalence of UIA in the study population. Reported p values were two sided, with significance set <0.05. Heterogeneity among included studies was assessed by χ^2^ statistics. For analysis, we used Review Manager (RevMan), V.5.3; Copenhagen: The Nordic Cochrane Centre, The Cochrane Collaboration, 2014.

### Standard protocol approvals, registrations and patient consents

Written informed consent or assent from relatives was obtained in all participants for study interview and follow-up, including ongoing review of primary care and hospital records and death certificate data. OXVASC was approved by the Oxfordshire research ethics committee (OREC A: 05/Q1604/70).

## Results

Of 2498 eligible patients referred (1143/45.8% TIA, 695/27.8% minor ischaemic stroke and 660/26.4% stroke mimics), 2013 underwent intracranial vascular imaging (1658/82.4% MRA and 355/17.6% CTA). Patients who did not receive intracranial vascular imaging (principally due to non-vascular diagnosis, dementia or patient refusal) were more likely to be female; have a background of atrial fibrillation, peripheral vascular or ischaemic heart disease and have a non-cerebrovascular discharge diagnosis (online supplemental table 1).

Of the 2013 patients with intracranial vascular imaging, 103 previously unknown, saccular, asymptomatic UIA were identified in 95 (4.7%) patients. Four non-saccular (fusiform) aneurysms and one previously coiled aneurysm were identified in four patients, but were not included in the analyses. No previously known, but untreated UIA, were identified.

Of the 103 UIAs, 40 (38.8%) were located in the anterior cerebral or anterior communicating arteries, 29 (28.2%) in the middle cerebral artery, 22 (21.4%) in the (intracranial) internal carotid artery and 12 (11.7%) in the posterior cerebral, posterior communicating or basilar arteries. Of the 103 UIAs, 60 (58.3%) were ≤3 mm, 24 (23.3%) were 4–6 mm and 19 (18.4%) were ≥7 mm in diameter (mean diameter/SD=4.2/2.3 mm). Patients with UIA were older than those without, more likely to be female and had a higher burden of hypertension (all p<0.05) (table 1). There were no differences in imaging modality in patients with and without UIA (table 1). In the 82 patients (86.3%) with definite TIA/ischaemic stroke and UIA, 9 (11.0%) were in the same territory as the presenting cerebrovascular event. In the 13 patients (13.7%) with ‘stroke mimics’ and UIA, four had a discharge diagnosis of migraine, one with syncope, two with microvascular neuropathy, one with peripheral radiculopathy, three were unclear and two had functional neurological symptoms.

**Table 1:** Baseline population characteristics stratified by the presence of asymptomatic unruptured intracranial aneurysm

Characteristic	Patients with intracranial vascular imaging (N=2013)		P value
	No UIA (N=1918)*	UIA (N=95)	
Mean age (SD)	67.2 (15.2)	70.4 (12.9)	**0.044**
Male sex (%)	979 (51.0)	30 (31.6)	**<0.0001**
Caucasian (%)	1799 (93.8)	91 (95.8)	0.66
Hypertension (%)	975 (50.8)	60 (63.2)	**0.019**
Diabetes mellitus (%)	239 (12.5)	12 (12.6)	0.96
Hyperlipidemia (%)	608 (31.7)	27 (28.4)	0.50
Current smoker (%)	265 (13.8)	19 (20.0)	0.09
Atrial fibrillation (%)	261 (13.6)	9 (9.5)	0.25
Any vascular disease† (%)	510 (26.6)	26 (27.4)	0.87
History of stroke or TIA (%)	263 (13.7)	16 (16.8)	0.36
PVD (%)	107 (5.6)	3 (3.2)	0.31
IHD (%)	224 (11.7)	8 (8.4)	0.33
Event type			
TIA (%)	970 (50.6)	52 (54.7)	0.28
Minor stroke (%)	557 (29.0)	30 (31.6)	
Other diagnosis (%)	391 (20.4)	13 (13.7)	
Imaging modality			
CTA (%)	339 (17.7)	16 (16.8)	0.51
MRA (%)	1579 (82.3)	79 (83.2)	

*Including 1 patient with previously treated ruptured intracranial aneurysm and 2 patients with non-saccular (fusiform) aneurysms.

†Vascular disease=prior ischaemic stroke/TIA, PVD or IHD.

CTA, computed tomography angiography; IHD, ischaemic heart disease; MRA, magnetic resonance angiography; PVD, peripheral vascular disease; TIA, transient ischaemic attack; UIA, unruptured intracranial aneurysm.

**Figure 1:**
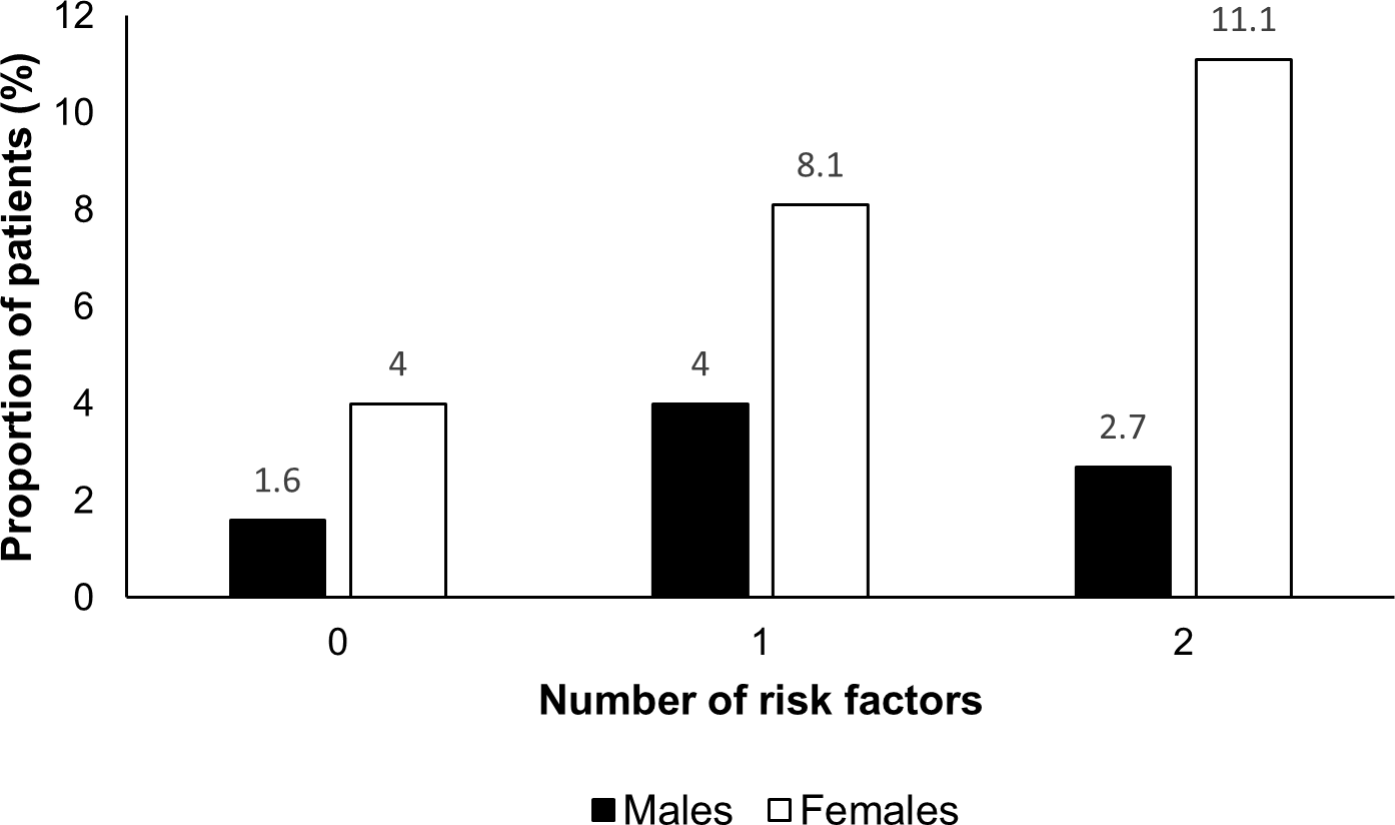
Prevalence of patients with unruptured intracranial aneurysms categorised by the number of risk factors (female sex, hypertension and current smoker) and sex.

Female sex, current smoking and hypertension were independent predictors of UIA (table 2). There was a significant trend of increasing prevalence of UIA with the presence of these risk factors (online supplemental figure 2); p_trend_ <0.0001). This was trend driven by female patients; if hypertensive and a smoker, the prevalence of UIA was 11.1% (figure 1). However, there was no difference in UIA size between male and female patients (mean/SD diameter 4.3/2.6 mm and 4.3/2.3 mm, respectively).

**Table 2:** Predictors of the presence of asymptomatic unruptured intracranial aneurysms

	Unadjusted risk indicators OR (95% CI)	P value	Age-adjusted risk indicators OR (95% CI)	P value	Multivariable risk indicators* OR (95% CI)	P value
Age (per 10 years)	1.15 (0.98 to 1.36)	0.083	–		1.13 (0.95 to 1.36)	0.18
Hypertension	1.66 (1.08 to 2.54)	0.020	1.54 (0.98 to 2.40)	0.060	1.59 (1.01 to 2.50)	**0.043**
Current smoker	1.56 (0.93 to 2.62)	0.093	1.20 (1.03 to 1.39)	0.021	2.07 (1.19 to 3.60)	**0.010**
Female sex	2.26 (1.45 to 3.51)	<0.0001	2.21 (1.42 to 3.44)	<0.0001	2.34 (1.50 to 3.66)	**<0.0001**

*The multivariable analyses includes each of the risk factors listed.

Other baseline characteristics stratified by the presence of UIA and discharge diagnosis are shown in online supplemental table 2. The prevalence of UIA was higher in patients with a discharge diagnosis of definite minor stroke/TIA (5.1%) compared with possible mimics (3.2%). Prevalence of UIA was unrelated to age (p_trend_ 0.18; online supplemental figure 3).

Two patients with UIA had a history of SAH from a different intracranial aneurysm. One patient had a family history of SAH (in a first-degree relative) and three patients had a possible family history of SAH (brain haemorrhage with unknown aetiology).

All scans with UIA were reviewed in a multidisciplinary meeting. Action was required in 44 (46.3%) patients (28/63.6% female): surveillance with interval scanning (n=37) and endovascular coiling (n=7). There were seven treated UIAs in those who underwent endovascular coiling: three middle cerebral artery, two basilar artery, one posterior communicating artery and one anterior communicating artery UIA, of mean size 7.3 mm (range 5–11 mm). Fifty patients (52.6%) were discussed at MDT but no follow-up imaging was required, either because they were considered low risk (n=40) or because the aneurysm or patient was considered too high risk for intervention (n=10).

Of the 95 patients with UIA, 1 (1.1%) had an SAH and 18 (18.9%) died of other causes during mean follow-up of 4.5 years (SD 2.4). One (1.1%) patient was lost to follow-up. Most identified UIAs (88.3%) had a predicted 5-year risk of rupture of less than 2% according to the PHASES risk score (table 3) and the overall average predicted risk was 0.9%. The observed 5-year risk of rupture in our cohort was 1.3% or 2.3 (95% CI 0.3 to 16.6) per 1000 person-years.

**Table 3:** Unruptured intracranial aneurysms in the OXVASC cohort stratified by 5-year risk of rupture calculated with PHASES score (taken from Greving *et al* 14)

PHASES score	Predicted 5-year risk of aneurysm rupture (95% CI)	Number of UIAs in OXVASC cohort, n=103 (%)
≤2	0.4% (0.1 to 1.5)	18 (17.5)
3	0.7% (0.2 to 1.5)	9 (8.7)
4	0.9% (0.3 to 2.0)	26 (25.2)
5	1.3% (0.8 to 2.4)	12 (11.7)
6	1.7% (1.1 to 2.7)	26 (25.2)
7	2.4% (1.6 to 3.3)	4 (3.9)
8	3.2% (2.3 to 4.4)	2 (1.9)
9	4.3% (2.9 to 6.1)	4 (3.9)
10	5.3% (3.5 to 8.0)	2 (1.9)
11	7.2% (5.0 to 10.2)	0 (0.0)
≥12	17.8 (15.2 to 20.7)	0 (0.0)

OXVASC, Oxford Vascular Study; PHASES, population, hypertension, age, size of aneurysm, previous subarachnoid haemorrhage (from a different aneurysm), site of aneurysm; UIA, unruptured intracranial aneurysm.

### Systematic review and meta-analysis

Nineteen studies comprising 12 781 patients with ischaemic stroke/TIA were identified in the systematic review (online supplemental figure 1). These were studies of either patients undergoing emergency investigation for acute major ischaemic stroke (n=12) or angiography for symptomatic carotid artery stenosis (n=7) (online supplemental table 3).

**Figure 2:**
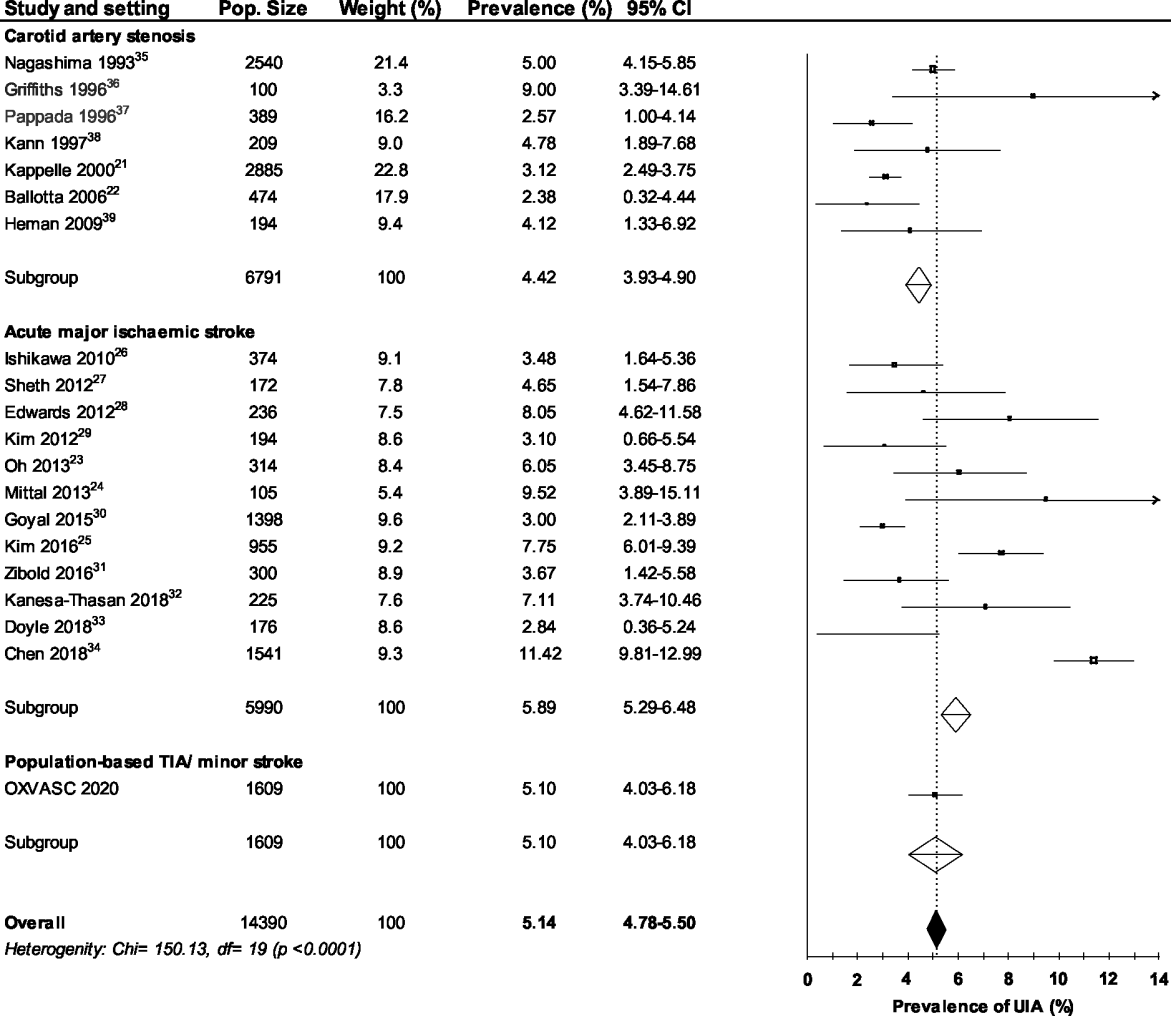
Forest plot depicting the prevalence of unruptured intracranial aneurysms in patients with ischaemic stroke or transient ischaemic attack (TIA), categorised by study selection criteria.^23–41^

The mean age of patients in all studies was 62.9 years (three studies did not report mean age of participants) and 41.6% of participants were female (five studies did not report the proportion of females). In studies of patients under investigation for symptomatic carotid artery stenosis (n=7), the mean UIA prevalence was 4.4% (95% CI 3.9 to 4.9). In studies of patients under investigation for acute major ischaemic stroke (n=12), the mean UIA prevalence was 5.9% (95% CI 5.3 to 6.5). The mean pooled UIA prevalence in all studies (including the OXVASC cohort patients with TIA/minor stroke) was 5.1% (95% CI 4.8 to 5.5, figure 2), but there was significant heterogeneity (p<0.00001).

Of the studies that reported UIA management (n=7), 80 patients with UIA (19.2%) underwent intervention. Only six studies provided sufficient information for the risk of subsequent SAH and length of participant follow-up (table 4); of these, there were four SAH in 217 patients over a mean follow-up of 2.5 years. The overall incidence of SAH (including the OXVASC data) was 4.6 (95% CI 1.9 to 11.0) per 1000 person-years.

**Table 4:** Risk of subarachnoid haemorrhage in patients with TIA and ischaemic stroke with UIA in studies identified in the systematic review

Study/setting	Recruitment period	Number of patients with UIA (%)	Person-years follow-up	Number of subsequent SAHs	Risk of SAH (per 1000 person-years)
Carotid artery stenosis					
Kappelle^23^	1988–1991	90 (3.1)	450.0	1	2.2
Ballotta^24^	1992–1999	11 (2.3)	55.0	0	0
Acute major ischaemic stroke					
Oh^25^	2007–2008	19 (6.1)	38.0	0	0
Mittal^26^	2011–2014	10 (9.5)	10.5	0	0
Kim^27^	2011–2014	74 (7.7)	111.0	3	27.1
Population-based TIA/minor stroke					
OXVASC 2020	2011–2020	95 (4.7)	427.5	1	2.3
Total	–	312 (5.3)	1092.3	5	4.6

SAH, subarachnoid haemorrhage; UIA, unruptured intracranial aneurysm.

## Discussion

In this first population-based study of patients with suspected TIA and minor stroke, we found an overall prevalence of asymptomatic UIA of 4.7%, increasing to 11.1% in hypertensive females who smoked. Although nearly half the patients found to have an UIA required additional investigations or procedures, there was only one SAH during follow-up. This is consistent with the overall predicted 5-year aneurysm rupture risk of 0.9% determined by the mean PHASES score.

UIA are formed by a combination of degenerative factors such as haemodynamic stress, a genetic predisposition and vascular risk factors, including hypertension, lipid accumulation and smoking.^16^ The largest meta-analysis to date of the prevalence of UIA included 95 000 individuals and reported an overall rate of 3.2%, higher in those with a background of atherosclerosis.^1^ Accordingly, our systematic review of UIA prevalence in patients with cerebrovascular disease demonstrated a UIA prevalence of 5.1%, although with significant heterogeneity. This is probably due to an excess of vascular risk factors, as demonstrated by patients with UIA in our cohort having similar baseline characteristics, irrespective of the discharge diagnosis, apart from a higher burden of hypertension in the non-stroke group (online supplemental table 2).

The significant heterogeneity in prevalence between previous studies is likely explained by the differing selection criteria of the acute ischaemic stroke studies. These studies variably selected consecutive patients with acute ischaemic stroke (n=6), or based on eligibility for thrombolysis (n=4) or thrombectomy (n=2). All studies were hospital based and ours is the only study to report prevalence and prognosis in patients with TIA and minor ischaemic stroke.

Risk factors for aneurysm growth and rupture are similar to those for UIA formation, with the addition of size and site.^16^ Most aneurysms identified in our cohort were estimated to be low risk which was consistent with the low rate of aneurysm rupture in our cohort. In addition, there may be protective anti-inflammatory effects of aspirin further reducing the risk of UIA rupture in these patients^5–7^; a clinical trial is ongoing (unique identifier: NCT02846259).^17^ There is a significant lack of prognostic data currently available—only six studies identified in the systematic review provided information regarding SAH risk and only two of these reported any outcome events (table 4). The power of our cohort was similar to the largest of these studies and demonstrated a similar risk of SAH: 2.3 and 2.2 per 1000 person-years, respectively.

The clinical implications of our findings are that clinicians should be aware of the higher prevalence of UIA in patients with cerebrovascular disease and emphasise risk factor control. Moreover, neuroradiologists should be attentive to the possible presence of UIA in patients with TIA/stroke, particularly females with other risk factors. Although there is insufficient evidence to suggest routine screening, the prevalence of UIA in female patients with minor stroke/TIA with other risk factors is higher than that of people in the general population with two or more first-degree relatives with SAH or UIA who are advised to undergo routine screening.^18^ Patients with TIA/stroke can be counselled that the overall risk of rupture is low during the next 5 years in the context of management according to American Heart Association/American Stroke Association guidelines for incidental aneurysms more generally, and might be lowered further by medication compliance and smoking cessation. A standard approach to UIA management appears to be applicable to this cohort.

The strengths of our study include its population-based nature with high rates of ascertainment and near complete investigation and follow-up. Patients were reviewed by a single senior neurologist and imaging was reported by a single neuroradiologist to maximise diagnostic consistency. More than 80% of eligible patients underwent intracranial vascular imaging, mostly MRA, which has a high sensitivity in detecting UIA and is comparable with that of CTA.^19^ Subsequent management of UIA reflected American Heart Association/American Stroke Association guidelines.

However, our study does have some limitations. First, some patients did not receive intracranial vascular imaging, but this was due to unavoidable issues (such as severe dementia, patient refusal or contraindication to contrast media) or patients who presented with unambiguous mimics (often with few vascular risk factors), and so our findings can still be applied to a typical neurovascular clinic setting. Second, although more than 80% of patients in our study received our first preference of MRI/MRA brain, CTA had to be used when MRA was contraindicated. However, there was no difference in the proportion of patients receiving either modality in the UIA and no UIA groups. Third, the UIA cohort was not powered to provide a precise estimate of prognosis. However, ours is the largest study so far on the prevalence and prognosis of incidental UIA in patients with cerebrovascular disease, although future studies with longer follow-up will be required to confirm the natural history UIA in this group. Fourth, although our results suggest that non-invasive angiography should be performed to screen for UIA in higher-risk patients, we are not able to conclude that this affects the risk of subsequent SAH and therefore improves patient outcome. Moreover, the psychological burden on patients who are found to have an incidental UIA, which may be small and will not require further investigation, should not be understated.^20^ Fifth, our study represents one centre’s management approach, and although it is consistent with international guidelines, monitoring and intervention might be more intensive in other healthcare systems. However, the prognosis of our patients was good despite less intensive monitoring than in some centres, suggesting that our findings are likely to be generalisable. Sixth, although there are some small studies suggesting a role of symptomatic arterial embolisation from UIA,^21 22^ we did not prospectively determine their potential role in the aetiology of the presenting ischaemic event in our study. However, as only 11% of UIAs were in the same arterial territory as the presenting event, this is likely to be a rare phenomenon. Seventh, we have only included patients with minor or transient events in this study and our findings are not translatable to patients with major ischaemic stroke. However, our cohort was selected based on the lack of data in these patients, the high rates of investigation enabling an accurate estimation of UIA prevalence and those in whom the identification of an UIA would have the greatest clinical implication.

In conclusion, the overall prevalence of UIA in a TIA and minor stroke cohort is probably higher than in the general population. However, in the context of intensively managed vascular risk factors and a standard policy for surveillance and intervention, the risk of rupture is low. These findings should be considered when counselling patients and in service design.

## Data Availability

Data are available on reasonable request. All data relevant to the study are included in the article or uploaded as online supplemental information. Requests for access to the data reported in this paper will be considered by the corresponding author.
