# Supplementary material for: Prevalence, predictors and prognosis of incidental intracranial aneurysms in patients with suspected TIA and minor stroke: a population-based study and systematic review
Source: J Neurol Neurosurg Psychiatry. 2020 Nov 4;92(5):542–8. doi: 10.1136/jnnp-2020-324418 (PMC8053340; doi:10.1136/jnnp-2020-324418)
Supplement: Supplementary data [file jnnp-2020-324418supp001.pdf]

Supplementary material

Table 1: Baseline population characteristics stratified by whether the patient underwent intracranial vascular imaging.

	**Intracranial vascular imaging (n= 2,013)**	**No Intracranial vascular imaging (n= 485)**	**p-value**
Mean age (SD)	67.4 (15.1)	66.8 (18.4)	0.45
Male sex (%)	1009 (50.1)	210 (43.3)	**0.007**
Caucasian (%)	1890 (93.9)	401 (89.1)	
Hypertension (%)	1033 (51.3)	223 (50.9)	0.88
Diabetes mellitus (%)	251 (12.5)	65 (14.9)	0.17
Hyperlipidaemia (%)	635 (31.5)	125 (28.7)	0.25
Current smoker (%)	284 (14.1)	56 (13.0)	0.53
Atrial fibrillation (%)	270 (13.4)	87 (19.9)	**0.001**
Any vascular disease[§] (%)	536 (26.6)	156 (35.9)	**<0.0001**
History of stroke or TIA (%)	279 (13.9)	70 (16.0)	0.25
PVD (%)	110 (5.5)	55 (12.6)	**<0.0001**
IHD (%)	232 (11.5)	72 (16.6)	**0.004**
Event type			
TIA (%)	1022 (50.8)	121 (24.9)	
Minor stroke (%)	587 (29.2)	108 (22.3)	**<0.0001**
Other diagnosis (%)	404 (20.1)	256 (52.8)	

PVD= peripheral vascular disease, IHD= ischemic heart disease, MRA= magnetic resonance angiography, CTA= computed tomography angiography.

[§]Vascular disease= prior ischemic stroke/ TIA, PVD or IHD.

Supplementary Table 2: Baseline population characteristics stratified by the presence of asymptomatic unruptured intracranial aneurysm and discharge diagnosis.

Characteristic	Patients with intracranial vascular imaging (n= 2,013)					
	**No UIA (n= 1,918)**			**UIA (n= 95)**		
	**Stroke/ TIA (n= 1,527)**	**Other diagnosis (n= 391)**	**p-value**	**Stroke/ TIA (n= 82)**	**Other diagnosis (n= 13)**	**p-value**
Mean age (SD)	69.0 (14.0)	60.4 (17.3)	<0.0001	71.2 (12.4)	65.2 (15.1)	0.12
Male sex (%)	802 (52.5)	177 (45.3)	0.010	28 (34.1)	2 (15.4)	0.18
Caucasian (%)	1441 (94.4)	358 (91.6)	0.046	79 (96.3)	12 (92.3)	0.45
Hypertension (%)	822 (53.8)	153 (39.1)	<0.0001	56 (68.3)	4 (30.8)	**0.009**
Diabetes mellitus (%)	201 (13.2)	38 (9.7)	0.066	10 (12.2)	2 (15.4)	0.75
Hyperlipidemia (%)	508 (33.3)	100 (25.6)	0.0040	22 (26.8)	5 (38.5)	0.39
Current smoker (%)	216 (14.1)	49 (12.5)	0.41	17 (20.7)	2 (15.4)	0.65
Atrial fibrillation (%)	228 (14.9)	33 (8.4)	0.001	9 (11.0)	0 (0)	0.21
Any vascular disease[§] (%)	408 (26.7)	102 (26.1)	0.80	24 (29.3)	2 (15.4)	0.30
History of stroke or TIA (%)	210 (13.8)	53 (13.6)	0.92	14 (17.1)	2 (15.4)	0.88
PVD (%)	88 (5.8)	19 (4.9)	0.49	3 (3.7)	0 (0)	0.48
IHD (%)	187 (12.2)	37 (9.5)	0.13	8 (9.8)	0 (0)	0.24
Imaging modality						
CTA (%)	247 (16.2)	92 (23.5)	0.001	15 (18.3)	1 (7.7)	0.69
MRA (%)	1280 (83.8)	299 (76.5)		67 (81.7)	12 (92.3)	

Supplementary Table 3: Studies identified by systematic review of the prevalence of unruptured intracranial aneurysms in ischaemic stroke or TIA patients.

Study	Location	Sample size	Cohort description	Mean age (years)	Females (%)	Imaging modality	UIA n (%)	UIA outcome
Nagashima 1993[1]	Japan	2540	Single centre, investigation of symptomatic CAS	-	-	CA	127 (5.0)	45 (35%) surgically clipped, 5 SAH at mean interval 5.6 years
Griffiths 1996[2]	UK	100	Single centre, investigation of symptomatic CAS	62	48	CA	9 (9.0)	5 (56%) surgically clipped
Pappada 1996[3]	Italy	389	Multi-centre, investigation of symptomatic CAS	67	20	CA	10 (2.6)	8 (80%) surgically clipped
Kann 1997[4]	USA	209	Single centre, investigation of symptomatic CAS	68	-	CA	10 (4.8)	-
Kappelle 2000[5]	USA	2885	Symptomatic CAS patients recruited to NASCET	66	30	CA	90 (3.1)	8 (9%) surgically clipped. 1 SAH during mean follow-up 5 years.
Ballotta 2006[6]	Italy	474	Single centre, investigation of symptomatic CAS	72	-	CA	11 (2.3)	No intervention and no SAH during mean follow-up 5 years
Heman 2009[7]	Netherlands	194	Single centre, investigation of symptomatic CAS	70	34	CTA	8 (4.1)	-
Ishikawa 2010[8]	Japan	374	Single centre, IS inpatients	70	38	MRA	13 (3.5)	No SAH in 3 months follow-up
Edwards 2012[9]	USA	236	Single centre, IS pre-thrombolysis work-up	-	-	CTA/ MRA	19 (8.1)	1 (5%) SAH 24 hours after thrombolysis
Kim 2012[10]	Korea	194	Single centre, thrombolysed IS patients	70	39	CTA/ MRA	6 (3.1)	-

Sheth 2012[11]	USA	172	Single centre, IS inpatients	62	53	CTA/ MRA	8 (4.7)	-
Mittal 2013[12]	USA	105	Single centre, IS pre-thrombolysis work-up	69	44	MRA/ CTA/ CA	10 (9.5)	No SAH in mean follow-up 18 months
Oh 2013[13]	Korea	314	Single centre, IS inpatients	66	39	CTA	19 (6.1)	No intervention. No SAH in mean follow-up 2 years
Goyal 2015[14]	International	1398	Multi-centre, IS pre-thrombolysis work-up	-	-	CTA/ MRA	42 (3.0)	-
Kim 2016[15]	Korea	955	Single centre, IS inpatients	65	39	MRA	74 (7.7)	8 (12%) patients surgically clipped. 3 SAH in mean follow-up 18 months.
Zibold 2016[16]	Germany	300	Single centre, IS patients receiving EVT	70	46	CA	11 (3.5)	-
Doyle 2018[17]	USA	176[a]	Single centre, IS inpatients	68	51	CTA	5 (2.8)	-
Kanesa-Thasan 2018[18]	USA	225	Single centre, IS endovascular therapy candidates	65	53	CTA	16 (7.1)	-
Chen 2018[19]	USA	1541	Single centre, IS inpatients	66	49	MRA	176 (11.4)	

UIA= unruptured intracranial aneurysm, CAS= carotid artery stenosis, IS= ischemic stroke, CA= catheter angiography, CTA= computed tomography angiography, NASCET= North American Symptomatic Carotid Endarterectomy Trial, SAH= subarachnoid hemorrhage; EVT= endovascular thrombectomy.
[a]patients with intracranial vascular imaging, demographics relate to whole cohort (n= 200).

Supplementary Figure 1: Flow diagram of systematic review inclusion/ exclusion for prevalence of unruptured intracranial aneurysms in TIA/ stroke patients and Ovid MEDLINE and Embase search terms.

Embase search terms

1. exp intracranial aneurysm/
2. (intracranial adj3 aneurysm).ti,ab.
3. berry aneurysm.mp.
4. saccular aneurysm.mp.
5. 1 or 2 or 3 or 4
6. Prevalence/
7. Prevalen*.ti,ab.
8. 6 or 7
9. 5 and 8

Ovid MEDLINE search terms

1. intracranial aneurysm/
2. (intracranial adj3 aneurysm).ti,ab.
3. berry aneurysm.mp.
4. saccular aneurysm.mp.
5. 1 or 2 or 3 or 4
6. Prevalence/
7. Prevalen*.ti,ab.
8. 6 or 7
9. 5 and 8

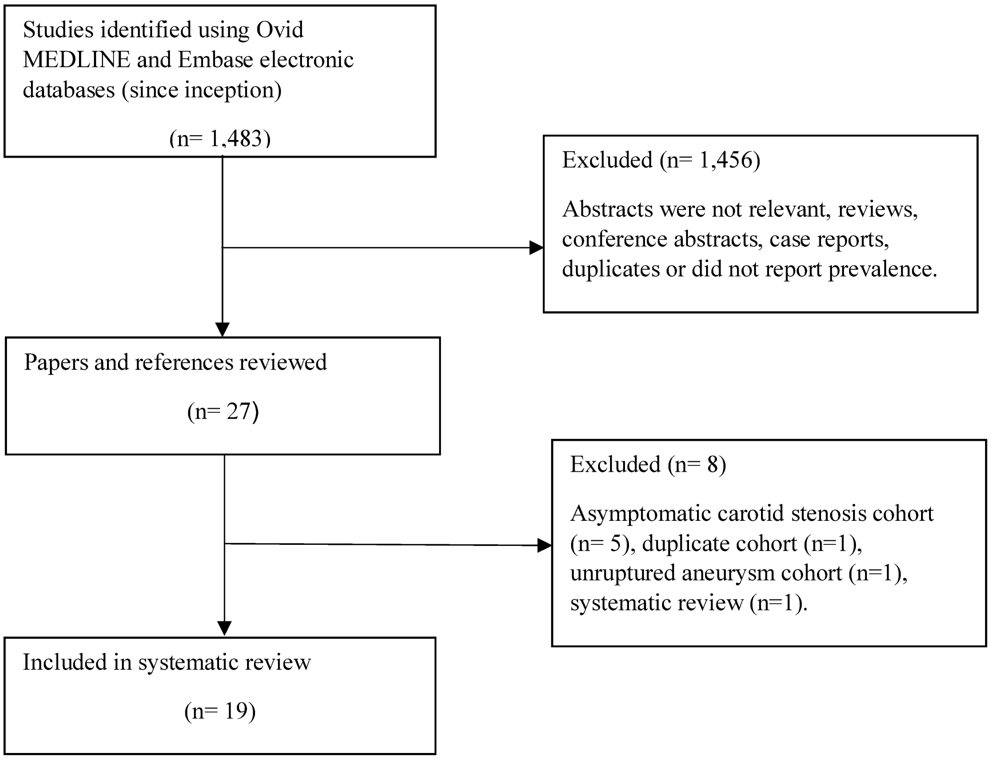

Supplementary Figure 2: Prevalence of patients with unruptured intracranial aneurysms categorized by the number of risk factors (female sex, hypertension and current smoker); p_{trend} <0.0001

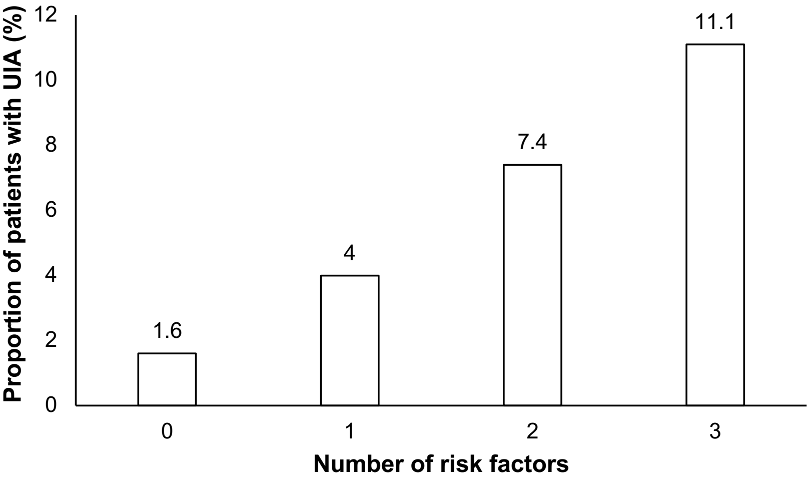

Supplementary Figure 3: Proportion of patients with UIA categories in 10-year age bands.

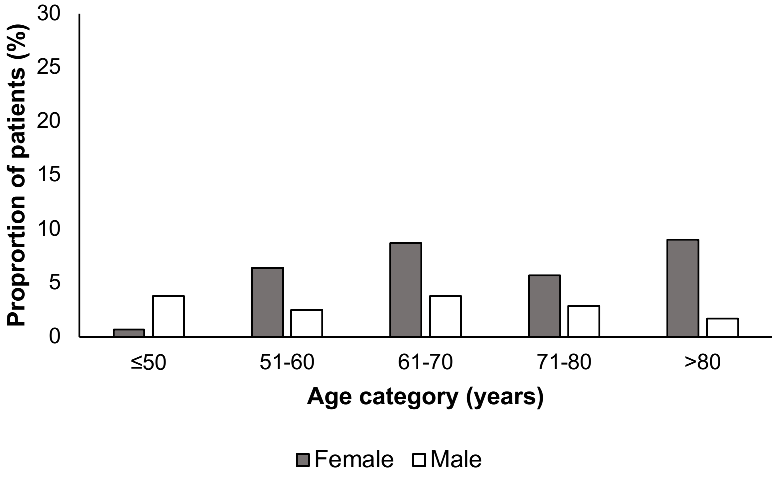

References

1. Nagashima M, Nemoto M, Hadeishi H, Suzuki A, Yasui N. Unruptured aneurysms associated with ischaemic cerebrovascular diseases. Surgical indication. Acta Neurochir (Wien). Austria; 1993;124:71–78.
2. Griffiths PD, Worthy S, Gholkar A. Incidental intracranial vascular pathology in patients investigated for carotid stenosis. Neuroradiology. Germany; 1996;38:25–30.
3. Pappada G, Fiori L, Marina R, Vaiani S, Gaini SM. Management of symptomatic carotid stenoses with coincidental intracranial aneurysms. Acta Neurochir (Wien). Austria; 1996;138:1386–1390.
4. Kann BR, Matsumoto T, Kerstein MD. Safety of carotid endarterectomy associated with small intracranial aneurysms. South Med J. United States; 1997;90:1213–1216.
5. Kappelle LJ, Eliasziw M, Fox AJ, Barnett HJ. Small, unruptured intracranial aneurysms and management of symptomatic carotid artery stenosis. North American Symptomatic Carotid Endarterectomy Trial Group. Neurology. United States; 2000;55:307–309.
6. Ballotta E, Da Giau G, Manara R, Baracchini C. Extracranial severe carotid stenosis and incidental intracranial aneurysms. Ann Vasc Surg. Netherlands; 2006;20:5–8.
7. Heman LM, Jongen LM, van der Worp HB, Rinkel GJE, Hendrikse J. Incidental intracranial aneurysms in patients with internal carotid artery stenosis: a CT angiography study and a metaanalysis. Stroke. United States; 2009;40:1341–1346.
8. Ishikawa Y, Hirayama T, Nakamura Y, Ikeda K. Incidental cerebral aneurysms in acute stroke patients: comparison of asymptomatic healthy controls. J Neurol Sci. Netherlands; 2010;298:42–45.
9. Edwards NJ, Kamel H, Josephson SA. The safety of intravenous thrombolysis for ischemic stroke in patients with pre-existing cerebral aneurysms: a case series and review of the literature. Stroke. United States; 2012;43:412–416.
10. Kim J-T, Park M-S, Yoon W, Cho K-H. Detection and significance of incidental unruptured cerebral aneurysms in patients undergoing intravenous thrombolysis for acute ischemic stroke. J Neuroimaging. United States; 2012;22:197–200.
11. Sheth KN, Shah N, Morovati T, Hermann LD, Cronin CA. Intravenous rt-PA is not associated with increased risk of hemorrhage in patients with intracranial aneurysms. Neurocrit Care. United States; 2012;17:199–203.
12. Mittal MK, Seet RCS, Zhang Y, Brown RDJ, Rabinstein AA. Safety of intravenous thrombolysis in acute ischemic stroke patients with saccular intracranial aneurysms. J Stroke Cerebrovasc Dis. United States; 2013;22:639–643.
13. Oh Y-S, Shon Y-M, Kim BS, Cho A-H. Long-term follow-up of incidental intracranial aneurysms in patients with acute ischemic stroke. J Stroke Cerebrovasc Dis. United States; 2013;22:329–333.
14. Goyal N, Tsivgoulis G, Zand R, et al. Systemic thrombolysis in acute ischemic stroke patients with unruptured intracranial aneurysms. Neurology. United States; 2015;85:1452–1458.
15. Kim JH, Suh SH, Chung J, Oh Y-J, Ahn SJ, Lee K-Y. Prevalence and Characteristics of Unruptured Cerebral Aneurysms in Ischemic Stroke Patients. J stroke. Korea (South); 2016;18:321–327.

16. Zibold F, Kleine JF, Zimmer C, Poppert H, Boeckh-Behrens T. Aneurysms in the target vessels of stroke patients subjected to mechanical thrombectomy: prevalence and impact on treatment. J Neurointerv Surg. England; 2016;8:1016–1020.

17. Doyle SJ, George BP, Holloway RG, Kelly AG. Incidental Findings in Radiographic Imaging for Inpatients with Acute Ischemic Stroke. J Stroke Cerebrovasc Dis. United States; 2018;27:3131–3136.

18. Kanesa-Thasan R, Cox M, Patel M, et al. Actionable vascular and other incidental findings on CTA in patients undergoing acute stroke intervention. Neuroradiol J. United States; 2018;31:572–577.

19. Chen ML, Gupta A, Chatterjee A, et al. Association Between Unruptured Intracranial Aneurysms and Downstream Stroke. Stroke. United States; 2018;49:2029–2033.
